# Nonsense-Mediated Decay Enables Intron Gain in *Drosophila*

**DOI:** 10.1371/journal.pgen.1000819

**Published:** 2010-01-22

**Authors:** Ashley Farlow, Eshwar Meduri, Marlies Dolezal, Liushuai Hua, Christian Schlötterer

**Affiliations:** Institut für Populationsgenetik, Veterinärmedizinische Universität Wien, Wien, Austria; National Institute of Genetics, Japan

## Abstract

Intron number varies considerably among genomes, but despite their fundamental importance, the mutational mechanisms and evolutionary processes underlying the expansion of intron number remain unknown. Here we show that *Drosophila*, in contrast to most eukaryotic lineages, is still undergoing a dramatic rate of intron gain. These novel introns carry significantly weaker splice sites that may impede their identification by the spliceosome. Novel introns are more likely to encode a premature termination codon (PTC), indicating that nonsense-mediated decay (NMD) functions as a backup for weak splicing of new introns. Our data suggest that new introns originate when genomic insertions with weak splice sites are hidden from selection by NMD. This mechanism reduces the sequence requirement imposed on novel introns and implies that the capacity of the spliceosome to recognize weak splice sites was a prerequisite for intron gain during eukaryotic evolution.

## Author Summary

The surprising observation 30 years ago that genes are interrupted by non-coding introns changed our view of gene architecture. Intron number varies dramatically among species; ranging from nine introns/gene in humans to less than one in some simple eukyarotes. Here we ask where new introns come from and how they are maintained in a population. We find that novel introns do not arise from pre-existing introns, although the mechanisms that generate novel introns remain unclear. We also show that novel introns carry only weak signals for their identification and removal, and therefore depend on nonsense-mediated decay (NMD). NMD maintains RNA quality control by degrading transcripts that have not been spliced properly. We propose that NMD shelters novel introns from natural selection. This increases the likelihood that a novel intron will rise in frequency and be maintained within a population, thus increasing the rate of intron gain.

## Introduction

Intron number is highly variable among eukaryotes, ranging from about a dozen in some fungi to more than 100,000 in the human genome. Comparative genomics across broad phylogenetic distances have identified the importance of both intron gain and loss to the establishment of this variation [1]. In particular for a number of lineages, including *Drosophila* [2], Caenorhabditis [3] and some isolated vertebrate lineages [4], a considerable number of intron gains have been described.

While there is a general agreement that the very first spliceosomal introns arose from the degeneration of self-splicing group II introns [5],[6], their complete absence from genomes that have undergone intron gain strongly suggests alternative mechanism(s) are at work. While several mechanism with varying levels of empirical support have been proposed over the last 30 years, there is still strong uncertainty over whether any existing model can explain the observed and predicted rates of intron gain throughout eukaryote evolution [7]. A satisfactory model must address the mutational mechanism that allows a intron to colonise a novel position and the evolutionary process that facilitates the fixation of this new allele within a population. An accounting of both mechanism and evolution should give insight into why the rate of intron gain is so variable between species.

Irrespective of the mutational mechanism, it is apparent that any new intron will require a number of key motifs including the 5′ and 3′ splice sites, and a set of auxiliary signals including the branch point and splicing enhancer and suppressor motifs [8],[9]. The failure to correctly identify an intron may either lead to stochastic alternative splicing or intron retention, both of which have deleterious consequences. This predicament is overcome if the newly inserted intron arrives fully functional. The only mechanism capable of generating a fully formed novel intron is reverse splicing [10],[11], in which an existing intron propagates into a new position, but this process is both extremely rare and inconsistent with the characteristics observed of novel introns [2]. The alternative is that novel introns develop gradually via the optimisation of previously non-intronic sequence. Examples include the intronisation of coding sequences [3], intron gain between paralogs of multi-copy gene families [4], the splicing of an Alu element [12], after internal gene duplication (including tandem duplication) [13] and after the insertion of new sequence of unknown origin [14].

In this study, we have investigated this alternative model in which novel introns are not required to be fully functional, relying instead on a back up mechanism of transcript quality control for incorrectly spliced introns [15]. In recent years it has become evident that the cell invests heavily in the identification of premature termination codons (PTCs) via the Nonsense Mediated Decay (NMD) pathway [16],[17]. NMD acts during the preliminary round of translation to identify in-frame stop codons and classify them as either genuine or premature. The use of incorrect splice sites or intron retention are a ready source of such premature termination codons (PTCs) and will invoke the NMD dependent destruction of the transcript.

Using comparative genomics of nine *Drosophila* species, we show that novel introns have weaker splice sites and carry more stop codons than conserved introns. We propose that NMD may play an important role during the establishment of novel introns within a population, and in support of this we identified a significant deficiency of novel introns that would remain invisible to the NMD pathway upon intron retention.

## Results/Discussion

**Figure 1:**
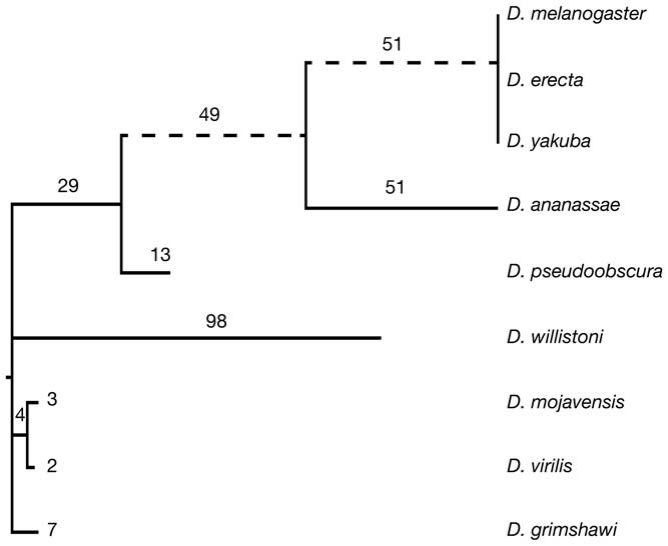
The uneven distribution of novel introns across *Drosophila* species. 307 novel introns were identified across a set of 3,593 genes with a full-length ortholog in each species. Dotted lines indicate branches with a greater number of novel than lost introns (Figure S2). Branch lengths are drawn proportionally to the rate of intron gain. The numbers of novel introns is indicated above each branch. 350 events occurred at the root of the tree and could not be classified as either intron gain or loss.

Here we have identified 307 novel introns amongst 284 genes across nine *Drosophila* genomes (Figure S1), presenting the most comprehensive set of novel introns to date. Our approach also detected 803 intron loss events amongst 595 genes, including 49 genes that have undergone both intron gain and loss (Dataset S1). These events show a strong heterogeneity across the *Drosophila* phylogeny, with several lineages being hot-spots of intron turnover (Figure 1 and Figure S2). We observe the highest rate of intron gain reported thus far, 2.8 intron gains/gene/Bya (10^9^) years in the *melanogaster* subgroup, being 6× greater than previously reported for *Drosophila* [2] and 4× greater than the next highest reported rate (occurring in yeast) [1],[18]. Interestingly, this rate is still higher than the range of estimates required to have generated the intron-rich eumetazoan genome (0.99–2.39 gains/gene/Bya years) [1],[19]. In sharp contrast, several other *Drosophila* lineages have experienced far less intron gain. *D. virilis* underwent only 0.0022 intron gains/gene/Bya years and since the split between *D. melanogaster* and *D. yakuba* 10 million years ago not a single intron gain could be identified, demonstrating that the rate of intron gain may vary over orders of magnitude between closely related species.

The previously proposed mechanisms of intron gain assume that new intronic sequence originates from elsewhere in the genome (reverse splicing [11],[20] or mobile elements [10],[12],[21]), or is derived from the endogenous genomic location (tandem duplication [22]–[25] or intronisation [3],[7]). Despite a rigorous search (Text S1) we could not identify an homologous parental origin for any novel intron elsewhere within the respective genomes, consistent with other studies [2],[26]. A manual inspection of the sequence flanking each novel intron identified a single event reminiscent of tandem duplication. The Bap170 gene in *D. pseudoobscura* has undergone a gain of 218 bp, of which only 206 bp are spliced out, revealing an imperfect 8 amino acid repeat 5′ and 3′ of this novel intron (Figure S3). While in final stage of preparing this manuscript Li *et al.*, (2009) reported that several novel introns in *Daphnia* are flanked by short direct repeats [14]. They insightfully suggest this may represent the signature of nonhomologous end joining (NHEJ) after uneven double-stranded breaks (DSBs), a process known to generate insertions flanked by direct repeats [27]. In consideration of this, we note that the duplication observed here may also be explained by a direct repeat flanking sequence of unknown origin. A manual inspection of dotplots identified 6 further examples in which direct repeats of length at least 8 bp overlapping the splice sites of a novel intron (Figures S4, S5, S6, S7, S8, S9) in support of the finding of Li *et al.*, (2009).

**Figure 2:**
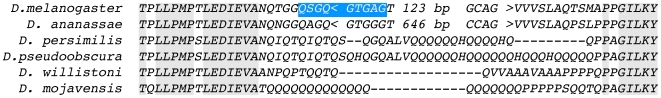
Intron gain in response to low complexity sequence in the gene CG42594. While the exact sequence of this highly variable region in the common ancestor of *D. melanogaster* and *D. ananassae* is not known, it is plausible that a single nucleotide deletion within the QSGQSG amino acid repeat (blue shading) generated the canonical 5′ splice site CAG | GTGAGT used by this phase 0 intron. Similarly, the CAG repeat (encoding poly-Q sequence) is a potent 3′ splice acceptor site [45]. Sequence conservation across all species is indicated with light shading. The novel intron (denoted by < >) is highly length variable across all species of the *melanogaster group*.

Reasoning that changes to the length of the coding sequence directly flanking a novel intron, as observed for Bap170, may give further insight into the mechanism of intron gain, we checked all 307 novel introns for alterations to the coding sequence that would indicate either the loss or gain of adjacent amino acids. Novel introns did not alter the ancestral coding sequence in 87% (267/307) of the cases. The remaining 13% (40/307) modified the adjacent coding sequence by only 1–3 amino acids (in 3 cases there was a gain of 4 or 5 amino acids along next to the new intron). This observation is inconsistent with the intronisation model of intron gain [3],[7] which requires the conversion of exonic sequence into an intron, hence reducing the coding sequence by the size of the new intron. A manual inspection of these 40 coding sequence-changing novel introns identified a small number of cases that can be explained via the conversion of low complexity amino acid sequence into an intron (Figure 2 and Figure S10). The novel intron within gene CG42594 has arisen from a rapidly evolving low complexity region including poly-Q sequence. Species lacking this intron show a highly variable sequence of amino acids at this position, with length differences of up to 18 amino acids. In the ancestor of *D. melanogaster* and *D. ananassae* this low complexity amino acid sequence was converted into an intron, stabilising the flanking protein sequence, while freeing the new intronic sequence of length constraint.

This indicates that the expansion of protein sequences can generate novel introns. Indels account for the majority of sequence variation between *Drosophila* species (3.2% of variable nucleotides vs. 1.8% for SNPs [28]) making them a significant contributor to both coding and non coding length evolution. Previous work focused on the mechanism underlying relatively short insertions (<15 bp), therefore, to access the possible contribution of exonic insertions to intron gain we identified insertions long enough to generate a novel intron (>44 bp in *Drosophila*). This revealed 180 insertions (Dataset S1), the largest being an insertion of 165 amino acids within the XNP gene of *D. pseudoobscura*. This demonstrates the plasticity of protein length and establishes large insertions within the protein coding sequence of *Drosophila* as a viable source of novel intronic sequence.

**Figure 3:**
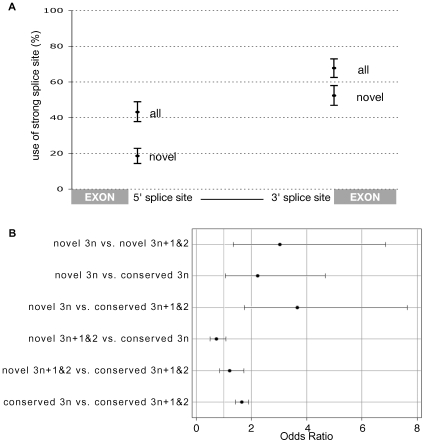
PTC as a backup for weak splice sites in novel introns. (A) The percentage of introns that use the most common 5′ and 3′ splice site motifs. Significantly fewer novel introns use the canonical GT(A/G)AGT motif at position +1 to +6 of the 5′ splice site. Likewise, fewer novel introns use CAG at −3 to −1 of the 3′ site. Error bars represent the 95% confidence intervals generated by resampling 307 introns with replacement 10,000 times (Figure S5). (B) A logistic regression identified a significant deficiency of 3n PTC-free introns within conserved introns (conserved 3n versus conserved 3n+1and2 - bottom contrast) confirming the finding of Jaillon *et al.* (2008) that selection acts against introns that would remain invisible to the NMD pathway upon intron retention. This effect is significantly stronger amongst novel introns (novel 3n versus novel 3n+1and2 - top contrast) and significantly stronger in a direct comparison between novel and conserved introns (second contrast) (95% CI that do not include one indicate a significant deficiency of 3n PTC-free introns).

We reason, that a much larger number of exonic insertions occur over evolutionary time providing the raw genetic variation for the gain of novel introns. The model that novel introns arise from a subset of “random” insertions within coding regions (or indeed UTR sequences) predicts that new introns are unlikely to arise with full strength splice sites. We observe that novel introns do in fact have weaker splice sites, with significantly reduced usage of the “strong” consensus motif at both the 5′ and 3′ splice site (Figure 3A and Figure S11). Furthermore, novel introns use a more diverse set of rare 5′ motifs than expected (Figure S11 and Dataset S1). Of course, weak, rare or atypical 5′ splice sites have lower affinity to the U1 snRNP of the spliceosome [29] which, all else being equal, leads to less efficient splicing [30],[31]. This poses a conundrum; if the mutational mechanism that generates novel introns leaves them vulnerable to suboptimal splicing, why do such novel introns rise to fixation within a population? We propose that the solution lies in the action of NMD.

Retention of 3n+1 and 3n+2 introns is expected to induce NMD due to the introduction of a frame-shift, but introns of length 3n require an in-frame PTC or they will remain invisible to the NMD pathway. Because of this, we reason that the failure to splice a new 3n insertion maybe deleterious, hence we predicted that novel 3n introns are more likely to encode a PTC as a backup mechanism for incomplete splicing. As the expectation for PTC occurrence is proportional to intron length, we fitted a logistic regression, modelling intron length, intron phase and a combined main effect of 3n class (3n vs. 3n+1 and 3n+2) and whether an intron is novel (n = 307) or conserved (n = 8,810) (Text S1). Despite its simplicity, our model was highly significant (P<0.0001) and explained 24% of the variation in the occurrence of stop codons among introns. Interestingly, most of the variation was explained by phase (Wald χ^2^ = 331.5, P<0.0001) and not intron length (Wald χ^2^ = 174.2, P<0.0001). Phase 2 introns encode significantly more in-frame PTCs than either phase 0 and 1 due to the sequence requirements of the 5′ splice site. The canonical 5′ splice site GT(A/G)A restricts the first full potential codon of a phase 2 intron to either the TAA Ochre or TGA Opal stop codon. Only a minority of introns with non-canonical splice sites escape this constraint.

Our analysis indicates that selection acts against introns that are invisible to the NMD pathway (if they undergo intron retention) leading to a deficiency of 3n PTC-free introns across the genome, as previously reported [32] (Figure 3B). This verifies in *Drosophila* that NMD carries a significant load caused by the weak splicing of introns [16]. We also observe this deficit of 3n PTC-free introns within the 307 novel introns. Interestingly, we find that this effect is significantly stronger among novel introns than among conserved introns (Odds ratio of 3.027 for novel vs. 1.646 for conserved), supporting the central role of NMD in the establishment of newly inserted sequence as novel introns.

**Figure 4:**
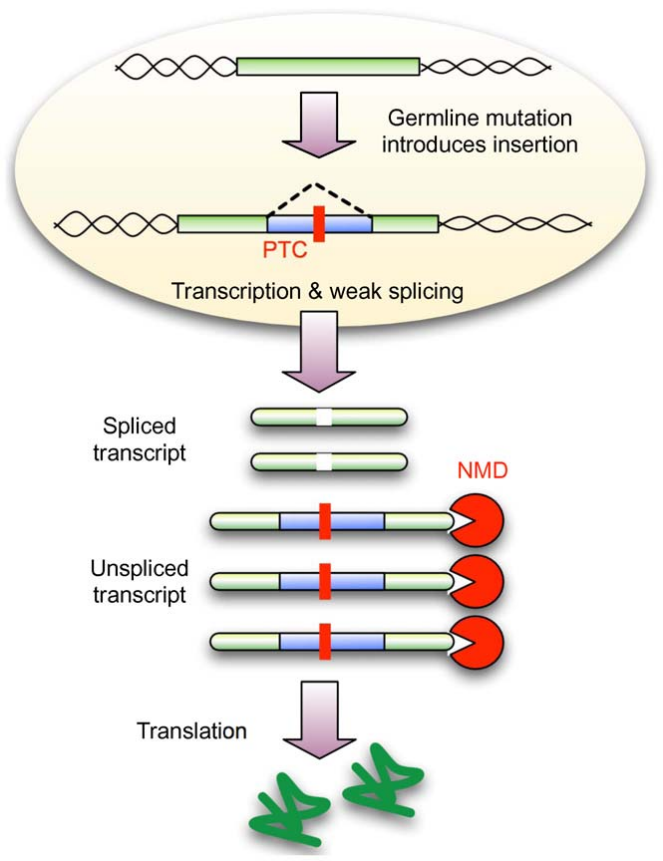
NMD conceals weakly spliced novel introns from selection. A new insertion in exonic sequence (or UTRs) that has the potential to undergo weak splicing but also disrupts the coding sequence (due to frame-shift or an in-frame PTC) will lead to a population of spliced and unspliced transcripts. NMD is expected to remove any unspliced transcript, leading to the translation of only the correct protein product. If sufficient protein is produced, the new insertion might be hidden from selection, thus allowing subsequent mutations to improve splicing and reducing the requirement for NMD. A new insertion that does not evoke NMD (3n PTC-free) will not enjoy this advantage and must encode strong splice sites from the beginning.

Here we have shown that while the expansion of amino acid repeats within exons can generate novel introns, nevertheless, the sequence origin for the vast majority remains unknown. This observation is inconsistent with previously suggested mechanisms of intron gain, but supported by the recent study of novel introns within *Daphnia* [14]. We have demonstrated that novel introns in *Drosophila* use weaker splice sites and are deficient for 3n PTC-free introns. Therefore, our evidence suggests that the establishment of these new sequences as introns is facilitated by NMD. Therefore, we propose a new model of intron gain (Figure 4), in which mutational mechanisms generate insertions that already carry the minimal requirements for correct, but not necessarily strong splicing. Cytoplasmic NMD is expected to degrade any unspliced transcript, leaving a proportion with the correct coding sequence. Conditional on adequate expression levels, this will shelter the new intron from selection allowing it to segregate within the population as a neutral polymorphism. Importantly, NMD allows new introns to utilise a more degenerate set of splice sites, thereby increasing the likelihood that any new sequence may become captured by a novel intron.

This model makes several predictions: First, novel introns are not required to pass through a protein coding intermediate stage (as would be expected from the intronisation of existing exonic sequence) and therefore, should not show codon usage bias. We observed no correlation between the “codon” usage of novel introns and the expected codon usage for *Drosophila* genes (Spearman Correlation Coefficient 0.01983, P = 0.8764) (Figure S12 and Text S1). Second, in general, introns with weaker splice sites are expected to suffer higher rates of failed splicing (intron retention or exon skipping), but we observe less intron retention among novel introns (2.6%) compared to conserved introns (5.3%). This is consistent with our expectation that via the action of NMD these transcripts are removed.

The “*faux* 3′ UTR” model suggests that PTC recognition depends on the distance to the downstream polyA tail [33],[34]. This makes NMD more potent towards the 5′ end of the transcript, leading to a third prediction; the establishment of novel introns should also be more efficient towards the 5′. As expected, we identified a strong and highly significant 5′ bias for novel introns (χ^2^ = 26.063, P<0.001) (Figure S13 and Text S1) in support of previous work [2]. NMD is more effective towards the 5′ as a PTC located towards the 3′ is more likely to be recognised as a canonical stop codon [7]. Hence, the involvement of NMD in the establishment of novel introns can explain the thus far enigmatic 5′ bias observed within a number of species [2],[7],[35]. The 803 lost introns reported here show no positional bias (χ^2^ = 1.309, P = 0.2526), consistent with previous reports [2],[18],[36]. In addition to 3′ UTR length the exon junction complex can invoke NMD in mammals. In effect this allows the recognition of PTCs in close proximity to the polyA tail, enhancing the effectiveness of NMD towards the 3′ of a transcript. Testing the influence of this on the distribution of novel introns is difficult due to their scarcity, but we note that mammalian genomes *do not* show the 5′ bias among all intron seen in *Drosophila* [35].

A significant question remains why does the rate of intron gain vary so much between closely related species? While differences in the action and potency of NMD are likely to exist between highly divergent taxa, we do not expect much variation on the fine scale of the *Drosophila* phylogeny. In contrast, the mutational processes that generate repeat expansions, tandem duplications [13], insertions of unknown origin [37] and DSBs are known to vary greatly between both closely and distantly related species. Differences in these underlying mechanisms will generate species specific variation upon which our proposed mechanism of intron fixation may act. This offers a possible explanation for the variation in intron gain rates observed here and over longer periods of eukaryotic evolution.

## Methods

### Discovery and validation of novel introns

Our approach to studying intron evolution is based on identifying gene orthologs across the *Drosophila* clade, predicting gene structure with GeneWise and using *Dollo Parsimony* to infer intron gain and loss events (Figure S1).

We identified orthologous genes using the *D. melanogaster* (release 4.3) gene set as the basis of a best-bidirectional-blast-hit approach in the 11 other sequenced *Drosophila* species, namely; *D. erecta*, *D. yakuba*, *D. ananassae*, *D. pseudoobscura*, *D. willistoni*, *D. virilis*, *D. mojavensis* and *D. grimshawi* (obtained from http://rana.lbl.gov/drosophila/). We excluded *D. sechellia*, *D. simulans and D. persimilis* because of low sequence coverage [38]. We acknowledge that a bidirectional-blast approach carries limitations but given our subsequent validation of intron turnover events feel this method was suitable. High-scoring segment pairs (HSPs) were identified via forward tblastx with default parameters followed by reverse tblastx using sequence cropped on either side of the best hit equivalent to the length of the corresponding gene in *D. melanogaster*. We considered the HSPs to be orthologous when the reverse blast identified only the parental gene in *D. melanogaster*.

Exon-intron structure of orthologous genes was generated by submitting to GeneWise [39] (2193 algorithm) the longest amino acid isoform of each *D. melanogaster* gene together with 100kb of nucleotide sequence flanking the corresponding orthologous hit. We excluded any gene with a frameshift mutation (either real or due to sequencing errors). Intron gain and loss events were predicted using the *Malin* java application [40]. The dense phylogeny of sequenced *Drosophila* genomes increases the power of *Dollo Parsimony* to accurately infer intron gain events, reducing the advantages of maximum likely methods [41]. Along two branches of the phylogeny (leading to *D. willistoni* and *D. grimshawi*) *Dollo Parsimony* remains sensitive to multiple losses being inferred as intron gain, but given the active debate about the best methods to infer intron turnover [42] we feel our approach and extensive downstream validation have proved reliable.

As our approach relies on de novo gene structure prediction via GeneWise it is sensitive to false positive and false negative intron prediction in other species. This problem was avoided in a previous study by considering only introns present in the well annotated *D. melanogaster* lineage [2]. Our approach takes full advantage of the multiple sequences genomes to find intron gain events outside of *D. melanogaster*, but required extensive validation to overcome the several limitations of GeneWise (detailed in Text S1 and Dataset S1).

This approach generated a high confidence set of 3,593 fully annotated orthologous genes (containing 8,810 introns) across nine *Drosophila* species, allowing us to identify intron gain and loss events across 40Mys of *Drosophila* evolution. Our approach is based on the amino acid sequence in *D. melanogaster* and is therefore not able to predict UTR introns. After this we still expected our data set to contain false positives (predicted novel introns that are not really introns) and false negatives (real introns that have been missed). Our experimental and informatic methods for their identification and exclusion are detailed in the Text S1. Novel intron sequences and gene, protein and intronic sequences for our orthologous gene set are available for download at http://i122server.vu-wien.ac.at/Drosophila_annotation/.

### The strength of novel splice sites

As per previous studies [43],[44], we used the percentage of introns with the consensus 5′ splice site GT(A/G)AGT (position +1 to +6) as a measure of the splice site strength within each class of introns. To confirm that novel introns use this motif significantly less than all introns we resampled (bootstrap with replacement) 307 introns from the population of 50,836 *D. melanogaster* introns 10,000 times (Figure S11A). The top and bottom 2.5% of samples gave the 95% confidence intervals on the observed percentages for all introns. The observed percentage of novel introns fell outside these confidence intervals establishing significance. Resampling (307 from 307, with replacement) from novel introns (black bars in Figure S11A and S11B) gives an indication of the variance within novel introns, but is not actually required to establish the significance between all and novel. We repeated this approach for the CAG motif at −3 to −1 of the 3′ splice site (Figure S11B). To show that novel introns use a more diverse set of rare/weak motifs at the 5′ we used the same bootstrap data from above and counted the number of different motifs present in each sample (Figure S11C).

## Supporting Information

Figure S1 Schematic of our approach and findings.
(0.26 MB EPS)

Figure S2 Intron loss rates across *Drosophila* species. Details as per Figure 1 in the text.
(0.06 MB EPS)

Figure S3 Duplication within the Bap170 gene (CG3274, FBgn0042085) of *D. pseudoobscura* associated with a novel intron. (A) Dotplot showing the subtle signal of direct repeats at either end of the novel intron. Window size = 8 bp, mismatch = 0. 50 bp of flanking exon are included. (B) Novel intron sequence (lower case) with the repeat underlined showing identity of 16/18 bp at the splice sites. The remaining intronic sequence finds no significant BLAST hit within NCBI. (C) Sequence alignment between three species showing that the gain of 218 bp resulted in an intron of only 206 bp, producing four novel amino acids in the 5′ exon. (D) A model showing that only four nucleotide substitutions within 48 bp are required to regenerate eight identical amino acids (LKLATTAT) at both ends of the intron.
(0.11 MB PDF)

Figure S4 A direct repeat of length 12/13 bp in the Histone deacetylase 3 gene of *D. ananassae* is associated with a novel intron of length 62 bp. (A) Dotplot with 50 bp of flanking exon. Window size = 8 bp, mismatch = 0. (B) Novel intron sequence (lower case) with the repeat (underlined) showing identity of 12/13 bp. The remaining intronic sequence finds no significant BLAST hit within NCBI. (C) Sequence alignment between three species.
(0.10 MB PDF)

Figure S5 A direct repeat of length 10/10 bp in the Autophagy-specific gene 9 (Atg9) gene of *D. virilis*. (A) Dotplot with 50 bp of flanking exon. Window size = 8 bp, mismatch = 0. (B) Novel intron sequence (lower case) with the repeat (underlined) and splice sites (bold). The remaining intronic sequence finds no significant BLAST hit within NCBI.
(0.04 MB PDF)

Figure S6 A direct repeat of length 8/8 bp in the CG2794 gene of *D. grimshawi*. (A) Dotplot with 50 bp of flanking exon. Window size = 8 bp, mismatch = 0. (B) Novel intron sequence (lower case) with the repeat (underlined) and splice sites (bold). The remaining intronic sequence finds no significant BLAST hit within NCBI.
(0.04 MB PDF)

Figure S7 A direct repeat of length 11/12 bp in the CG3295 gene of *D. willistoni*. (A) Dotplot with 50 bp of flanking exon. Window size = 8 bp, mismatch = 0. (B) Novel intron sequence (lower case) with the repeat (underlined) and splice sites (bold). The remaining intronic sequence finds no significant BLAST hit within NCBI.
(0.04 MB PDF)

Figure S8 A direct repeat of length 11/12 bp (or maybe 14/17) in the CG9536 gene of *D. willistoni*. (A) Dotplot with 50 bp of flanking exon. Window size = 8 bp, mismatch = 0. (B) Novel intron sequence (lower case) with the repeat (underlined) and splice sites (bold). The 5′ and 3′ splice sites are not within the direct repeat, but in close proximity. The remaining intronic sequence finds no significant BLAST hit within NCBI.
(0.04 MB PDF)

Figure S9 A direct repeat of length 8/8 bp in the CG5181 gene of the melanogaster subgroup. (A) Dotplot with 50 bp of flanking exon. Window size = 8 bp, mismatch = 0. (B) This novel introns was gained in the ancestor of mel, ere and yak, the sequence here is taken for the novel intron of *D. yakuba* (lower case) with the repeat (underlined) and splice sites (bold). The remaining intronic sequence finds no significant BLAST hit within NCBI.
(0.05 MB PDF)

Figure S10 A novel intron within the gene CG34382 has captured only part of the low complexity sequence. (A) The poly-Q region of the 5′ exon has continued to undergo length change in species with the novel intron. (B) The exon-2/intron-2 boundary from *D. melanogaster*. The flanking exonic sequence contains an imperfect CAG repeat, which is not present within the novel intron. This “new” intron pre-dates the split of *D. melanogaster* and *D. ananassae* and is therefore at least 14 million years old, sufficient time for any repeat structure to break down within non-coding sequence. (* indicate conserved amino acids.)
(0.02 MB PDF)

Figure S11 Resampling analysis of splice site usage. We resampled (with replacement) 307 from the set of 307 novel introns (black) and 307 from the set of all 50,836 introns (gray) to obtain a distribution of the proportion of introns that carry the most common motif at the 5′ (A) and at the 3′ (B). The observed values for novel (black dot) and all (gray dot) are shown below each graph with 95% CI taken from the distributions above. The observed values for novel are outside the 95% CI for the distribution for all introns. Resampling from the set of novel is not actually required to establish significance, but does give an indication of the variation within novel introns. (C) The 307 novel introns use 83 different motifs at the 5′ splice site (black dot), outside the distribution of values obtained by resampling (307 samples, 10,000 times) from all (393 different motifs, 50,836 introns), indicating that novel introns use a more diverse set of splice sites than expected (i.e. more rare/weak motifs).
(0.04 MB PDF)

Figure S12 Novel introns show not codon usage bias. Distribution of codon usage values for all 64 codons for (A) all *D. melanogaster* genes (http://www.kazusa.or.jp/codon/cgi-bin/showcodon.cgi?species=7227) (B) the 180 insertions excluded form our set of novel introns and (C) the 307 novel introns. Spearman Correlation Coefficients indicate significant codon usage bias in insertions (0.57989, P<0.0001) further justifying their exclusion from our data set, but no bias within novel introns (0.01983, P = 0.8764).
(0.08 MB PDF)

Figure S13 Novel introns are strongly biased towards the 5′ end of the gene. Empirical cumulative distribution of intron position across the gene for (A) novel and conserved introns, and (B) lost introns and insertions. Compared to a uniform distribution novel (X^2^ = 26.063, P<0.001) and conserved (X^2^ = 110.554, P<0.0001) both show a 5′ bias. This bias is stronger for novel introns (X^2^ = 7.273, P = 0.007). Lost introns (X^2^ = 1.309, P = 0.253) and insertions (X^2^ = 0.495, P = 0.482) do not differ from the uniform distribution.
(0.04 MB PDF)

Text S1 Supplemental methods.
(0.06 MB DOC)

Dataset S1 Includes Table S1, S2, S3, S4, S5, S6, S7, S8. Table S1. List of 3593 genes for which we identified a full length ortholog in all nine Drosophila species. Table S2. 307 novel introns, including length, length class, phase, PTC, and EST support. Table S3. 810 intron loss events. Table S4. 12 cases of concurrent intron gain and loss and cases of independent events. Table S5. 180 large protein coding insertions that do not undergo splicing (GeneWise false positives - exons predicted as novel introns). Table S6. 5′ and 3′ splice site usage for novel and control introns. Table S7. BLAST results against the EST data base. Table S8. 86 cases of failed intron prediction (GeneWise false negatives) in which the underlying intronic sequence and splice sites are still present, but GeneWise failed to predict an intron.
(0.65 MB XLS)
