# Supplementary material for: Nonsense-Mediated Decay Enables Intron Gain in Drosophila
Source: PLoS Genet. 2010 Jan 22;6(1):e1000819. doi: 10.1371/journal.pgen.1000819 (PMC2809761; doi:10.1371/journal.pgen.1000819)
Supplement: Figure S3 — Duplication within the Bap170 gene (CG3274, FBgn0042085) of D. pseudoobscura associated with a novel intron. (A) Dotplot showing the subtle signal of direct repeats at either end of the novel intron. Window size = 8 bp, mismatch = 0. 50 bp of flanking exon are included. (B) Novel intron sequence (lower case) with the repeat underlined showing identity of 16/18 bp at the splice sites. The remaining intronic sequence finds no significant BLAST hit within NCBI. (C) Sequence alignment between three species showing that the gain of 218 bp resulted in an intron of only 206 bp, producing four novel amino acids in the 5′ exon. (D) A model showing that only four nucleotide substitutions within 48 bp are required to regenerate eight identical amino acids (LKLATTAT) at both ends of the intron. (0.11 MB PDF) [file pgen.1000819.s003.pdf]

A

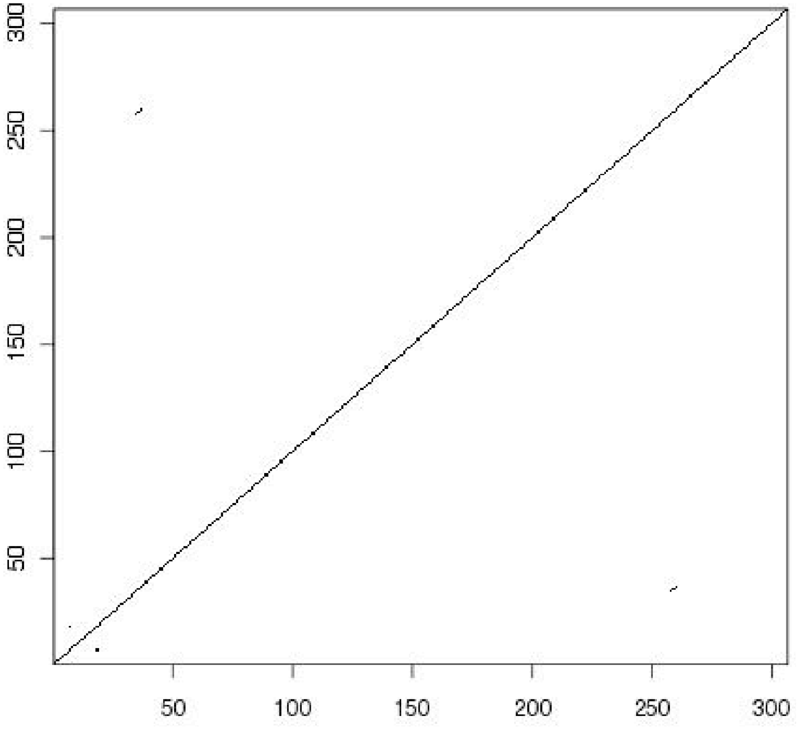

B

>FBgn0037792|2R|CG3274|Bap170-PA|pse(16/18 direct repeat)
GCAGTGGCTCCTCCCCGCTCCTCCACCACAGC**TCAAGCTGGC**C**A**A**CACGg**taagtg
cgttttggaatactcttaataccggtagcttctctatttgggtgggctcgcaatgag
cctgttctgtttggttgtgagaatagagtaggatcagatcccctgctgtagcgtcgg
cgaaaaatttcttccccattggtttttaactttaagtggtcactattgcaccgattc
atgatattatataatatatattt**tcaagCTGGC**T**A**C**CACGG**CCACCATCAAGACGGA
CCAGGGCCTACAGACGGTGCC

C

mel SPAPHSQLK-----------------------LATTATIKTD
pse SPAPPPQLK**LANT** GTA AGT//TTC AAG LATTATIKTD
moj APAPPPQLK-----------------------LATTATIKTD
*** *** **********

D

PPPQLKLATTATIK

↙ ↘

PPPQLKLATTAT...LKLATTATIK

↓

PPPQLKLA**N**T<G**T**A A**G**T...TT**C** AAG>LATTATIK
