# Supplementary material for: Nonsense-Mediated Decay Enables Intron Gain in Drosophila
Source: PLoS Genet. 2010 Jan 22;6(1):e1000819. doi: 10.1371/journal.pgen.1000819 (PMC2809761; doi:10.1371/journal.pgen.1000819)
Supplement: Figure S4 — A direct repeat of length 12/13 bp in the Histone deacetylase 3 gene of D. ananassae is associated with a novel intron of length 62 bp. (A) Dotplot with 50 bp of flanking exon. Window size = 8 bp, mismatch = 0. (B) Novel intron sequence (lower case) with the repeat (underlined) showing identity of 12/13 bp. The remaining intronic sequence finds no significant BLAST hit within NCBI. (C) Sequence alignment between three species. (0.10 MB PDF) [file pgen.1000819.s004.pdf]

A

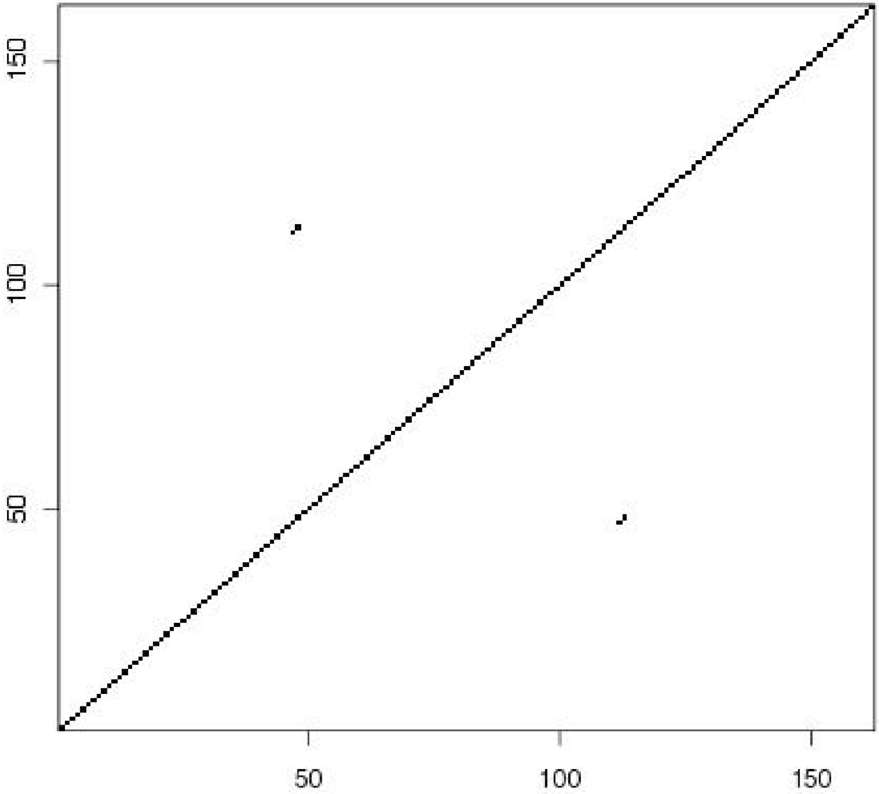

B

>FBgn0025825|3R|CG2128|438|Hdac3-PA|239|Ana
GCGTGAACGTCCCTCTTAAAGAAGGAATCGATGATCAGAGCTACTTCCAG**gt**gggta
tatatgaaccgccaagttgcttaggctactaatttgtactttcttctatttcc**ag**GT
GTTTAAACCCATTATTTCGGCCATCATGGACTTCTATCGACCTACTGC

C

```
>mel GATGACCAGAGCTATTTTCAG-----------------------GTGTTCAAACCCATAATTTC
>ana GATGATCAGAGCTACTTCCAGgtgggtata//ttctatttccagGTGTTTAAACCCATTATTTC
>pse GACGATCAGAGCTACTTCCAG-----------------------GTCTTCAAACCCATCATCTC
     ** ** ******** ** ***                       ** ** ******** ** **
```
