# Supplementary material for: Nonsense-Mediated Decay Enables Intron Gain in Drosophila
Source: PLoS Genet. 2010 Jan 22;6(1):e1000819. doi: 10.1371/journal.pgen.1000819 (PMC2809761; doi:10.1371/journal.pgen.1000819)
Supplement: Figure S5 — A direct repeat of length 10/10 bp in the Autophagy-specific gene 9 (Atg9) gene of D. virilis. (A) Dotplot with 50 bp of flanking exon. Window size = 8 bp, mismatch = 0. (B) Novel intron sequence (lower case) with the repeat (underlined) and splice sites (bold). The remaining intronic sequence finds no significant BLAST hit within NCBI. (0.04 MB PDF) [file pgen.1000819.s005.pdf]

A

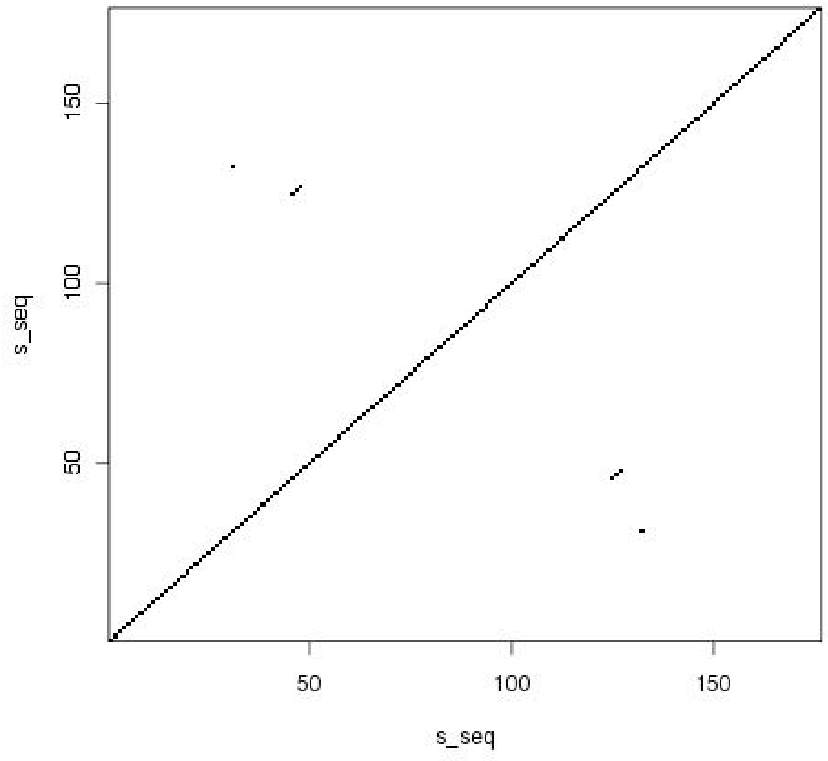

B

>FBgn0034110|2R|CG3615|Atg9-PA|Vir (10/10 direct repeat)
ACCAGCAGAAGCATGGCTTTCGTGTCATCGTGCTGGACGAGGTGTTTCAG**gt**gagcg
tcaaccgaactgcactttgaataaaatctccaagttgaaggcaaatgatttaaacca
actccgtttc**ag**GTGCTGGAATTCGGGTTTGTTGTCTGGCTGTTTACGTTTACGACG
CATTG
