# Supplementary material for: Nonsense-Mediated Decay Enables Intron Gain in Drosophila
Source: PLoS Genet. 2010 Jan 22;6(1):e1000819. doi: 10.1371/journal.pgen.1000819 (PMC2809761; doi:10.1371/journal.pgen.1000819)
Supplement: Figure S6 — A direct repeat of length 8/8 bp in the CG2794 gene of D. grimshawi. (A) Dotplot with 50 bp of flanking exon. Window size = 8 bp, mismatch = 0. (B) Novel intron sequence (lower case) with the repeat (underlined) and splice sites (bold). The remaining intronic sequence finds no significant BLAST hit within NCBI. (0.04 MB PDF) [file pgen.1000819.s006.pdf]

A

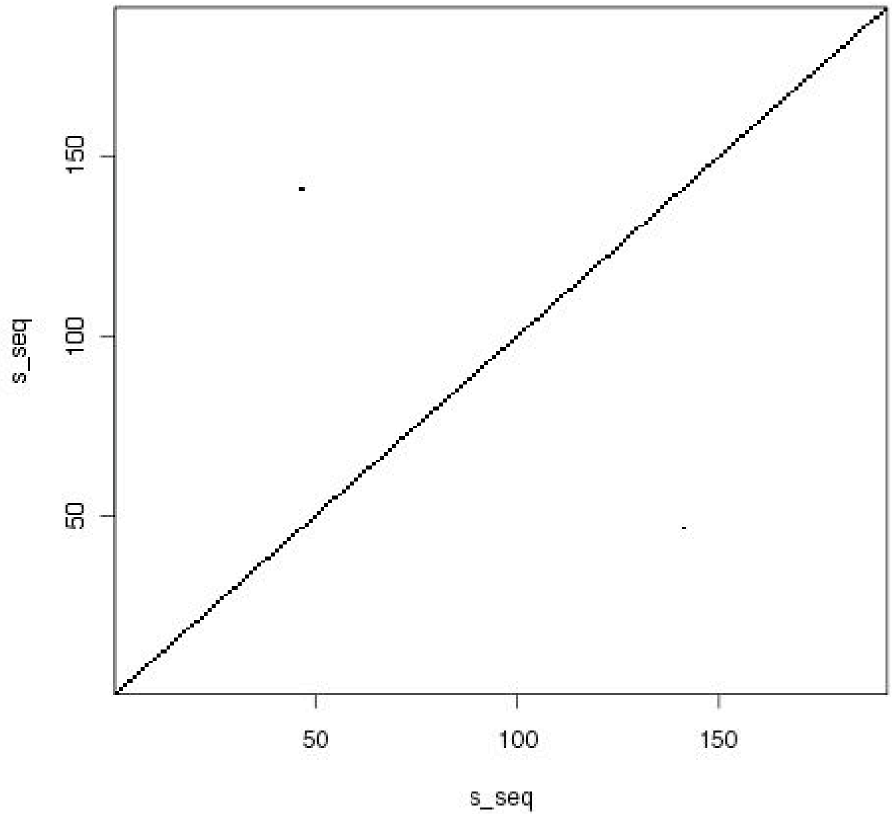

B

>FBgn0031265|2L|CG2794|CG2794-PA|gri (8/8 direct repeat)
TATCGGAGCAGGCAATGGCCTGCACACTGCAGCTGGCCCAGAAGTTTCAG**gt**aaagc
gaaccgaactctaaaataatacttagtttgaaattggaaaacgatgtttaaaaaata
aaaatcaataaacaactttcttttc**ag**GTGCCTGTGTTCTTTGAGCCCACGGATATG
TATATAGCTGGCAAACCGTT
