# Supplementary material for: Nonsense-Mediated Decay Enables Intron Gain in Drosophila
Source: PLoS Genet. 2010 Jan 22;6(1):e1000819. doi: 10.1371/journal.pgen.1000819 (PMC2809761; doi:10.1371/journal.pgen.1000819)
Supplement: Figure S8 — A direct repeat of length 11/12 bp (or maybe 14/17) in the CG9536 gene of D. willistoni. (A) Dotplot with 50 bp of flanking exon. Window size = 8 bp, mismatch = 0. (B) Novel intron sequence (lower case) with the repeat (underlined) and splice sites (bold). The 5′ and 3′ splice sites are not within the direct repeat, but in close proximity. The remaining intronic sequence finds no significant BLAST hit within NCBI. (0.04 MB PDF) [file pgen.1000819.s008.pdf]

A

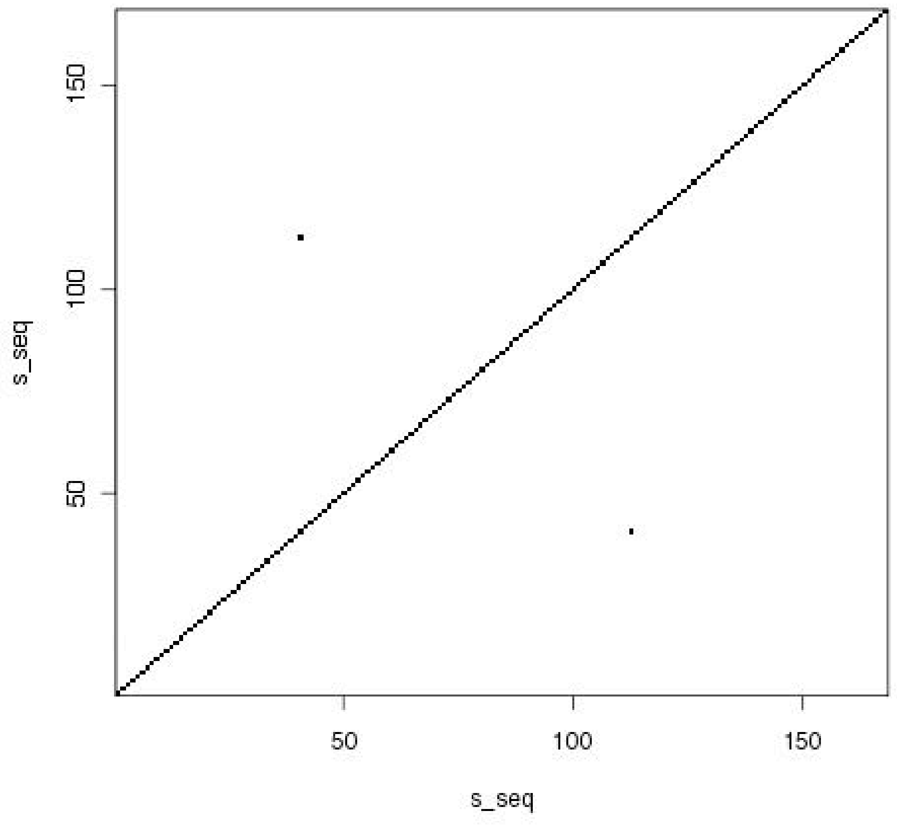

B

>FBgn0031818|2L|CG9536|CG9536-PA|wil (11/12 or 14/17 DR)
GGTTGTGGACCGCGTTCACATTTTGGCTTATTGAACTGCATTGGTGGAG**gt**atgga
tttgtgtatattttgctaaaagttaatgaattcctactatgattccacttcattggt
ga**ag**GTGGCTGTTGACGTTGTCACCGTTGGACTATGTGGAAAGATGCTGGAGCC
