# Supplementary material for: Nonsense-Mediated Decay Enables Intron Gain in Drosophila
Source: PLoS Genet. 2010 Jan 22;6(1):e1000819. doi: 10.1371/journal.pgen.1000819 (PMC2809761; doi:10.1371/journal.pgen.1000819)
Supplement: Figure S9 — A direct repeat of length 8/8 bp in the CG5181 gene of the melanogaster subgroup. (A) Dotplot with 50 bp of flanking exon. Window size = 8 bp, mismatch = 0. (B) This novel introns was gained in the ancestor of mel, ere and yak, the sequence here is taken for the novel intron of D. yakuba (lower case) with the repeat (underlined) and splice sites (bold). The remaining intronic sequence finds no significant BLAST hit within NCBI. (0.05 MB PDF) [file pgen.1000819.s009.pdf]

A

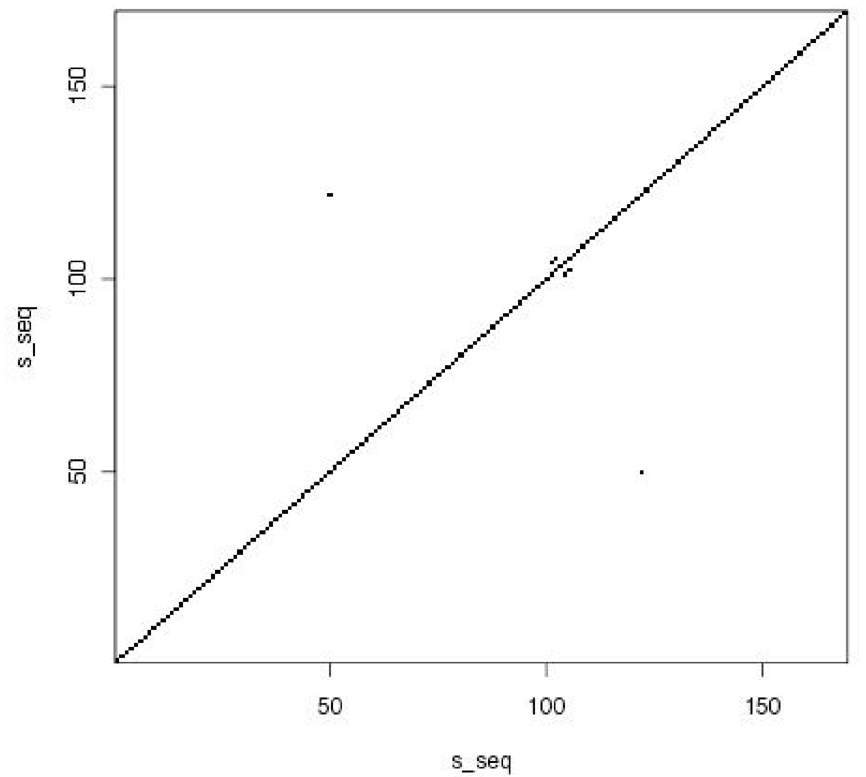

B

>FBgn0031909|2L|CG5181|CG5181-PA|gained in mel, ere & yak (sequence from yak)(8/8 direct repeat)

TGGCGTGGCCACCGTTACAAAGGAAAACCGCGAAGTGCGGAACTTTAAG**gt**gggtg
cactgttgcaatagtcgcataaaaacaatgaataactattaataataataaacattt
tca**ag**GTGGGCGATCCCACGGCCTGCATCAATGTCTCCATTTGGGACGAGCCAGG
