# Supplementary material for: Nonsense-Mediated Decay Enables Intron Gain in Drosophila
Source: PLoS Genet. 2010 Jan 22;6(1):e1000819. doi: 10.1371/journal.pgen.1000819 (PMC2809761; doi:10.1371/journal.pgen.1000819)
Supplement: Figure S10 — A novel intron within the gene CG34382 has captured only part of the low complexity sequence. (A) The poly-Q region of the 5′ exon has continued to undergo length change in species with the novel intron. (B) The exon-2/intron-2 boundary from D. melanogaster. The flanking exonic sequence contains an imperfect CAG repeat, which is not present within the novel intron. This “new” intron pre-dates the split of D. melanogaster and D. ananassae and is therefore at least 14 million years old, sufficient time for any repeat structure to break down within non-coding sequence. (* indicate conserved amino acids.) (0.02 MB PDF) [file pgen.1000819.s010.pdf]

A

```
D.mel GPYQLPLPLPAPQHRSVNPQQQQQHQQ<   61nt  >PYQVIPEEQFLKILEEELQ
D.sec GPYQLPLPLPAPKHRSVNPQQQQQQQHQQ< 61nt  >PYQVIPEEQFLKILEEELQ
D.sim GPYQLPLPLPAPQHRSVNPQQQQQQQQHQQ<61nt  >PYQVIPEEQFLKILEEELQ
D.yak GPYQLPLPLPAPQHRSVNPQQQQQQHQQ<  61nt  >PYQVIPEEQFLKILEEELQ
D.ere GPYQLPLPLPAPQHRLVNPQQQQQNQQ<   61nt  >PYQVIPEEQFLKILEEELQ
D.ana GPYQLPLPLPAPQHQPGNPQQQQPQQ<    60nt  >PYQIIPEEQFLKILEEELQ
D.pse GPYQLPLPLPAPQHQQGHPQQHQHQHQSQQ---------PYQVIPEEQFLKLLEEELQ
D.wil GPYQLPLPLPAPQHHQSQGHQQQQQQGSTQQ-----QQQPYQVIPEEQFLKLLEEELQ
D.vir GPYQLPLPLPAPQHQHQQQQHQQQQQ---------QQPYQIIPEEQFLKLLEEELQ
D.moj QAYQLPLPLPAPHHQHQQQQHPQQQQ----------QQYQIIPEEQFLKILEEELQ
D.gri VPYQLPLPLAAPQHQHRPQQQQQHQHQHQQQQQHAQQPYQIIPEEQFLKLLEEELQ
        ******* ** *                         ** ***************
```

B

```
CCC CAG CAG CAG CAG CAG CAA CAT CAG CAG  < GTGGGTCATCTAAATGC
GACGCTATAAAGCTCTTATACTAATCAATCCTACTTTCCAAAAG >
```
