# Supplementary figures and images for: Nonsense-Mediated Decay Enables Intron Gain in Drosophila
Source: PLoS Genet. 2010 Jan 22;6(1):e1000819. doi: 10.1371/journal.pgen.1000819 (PMC2809761; doi:10.1371/journal.pgen.1000819)

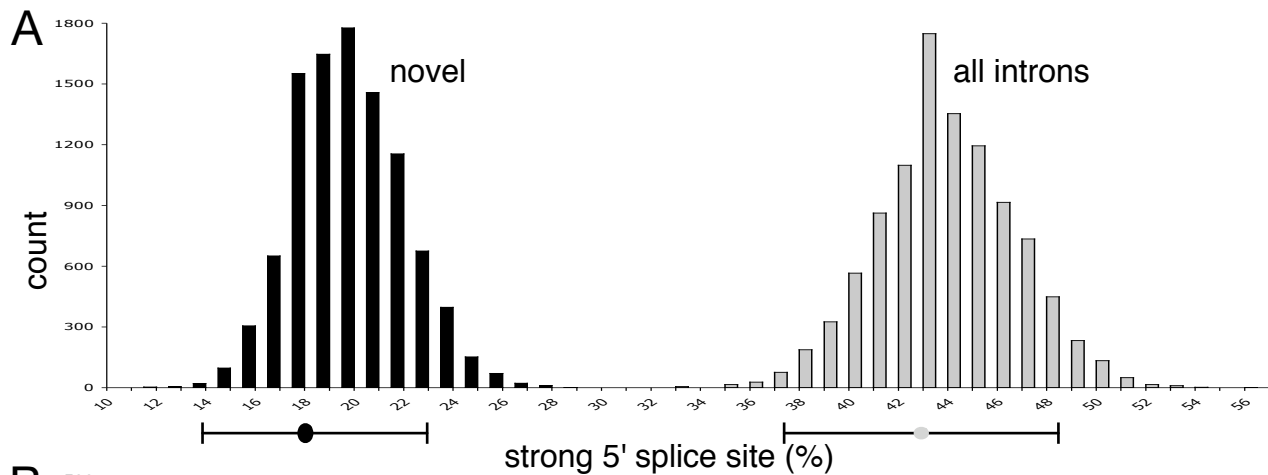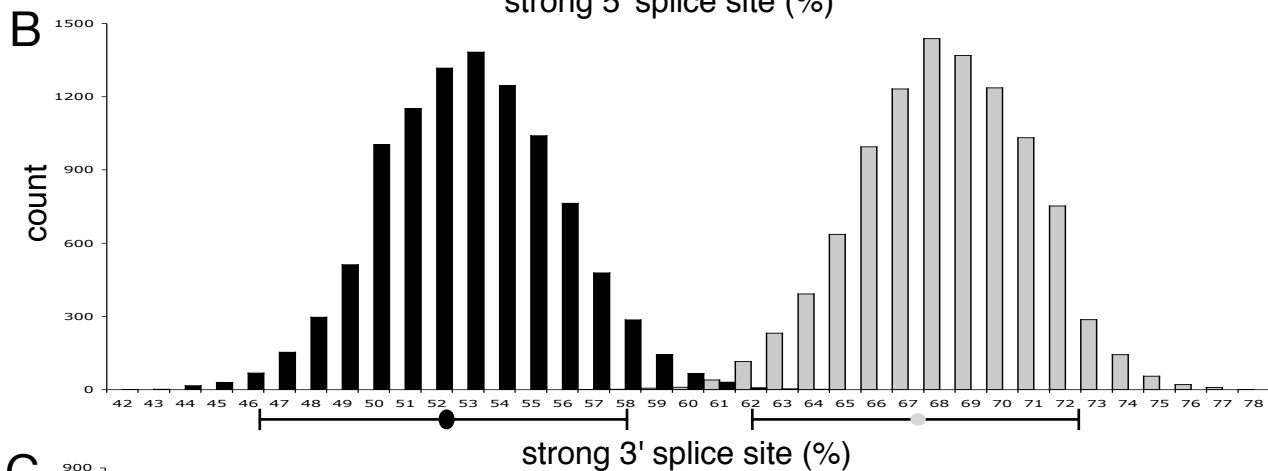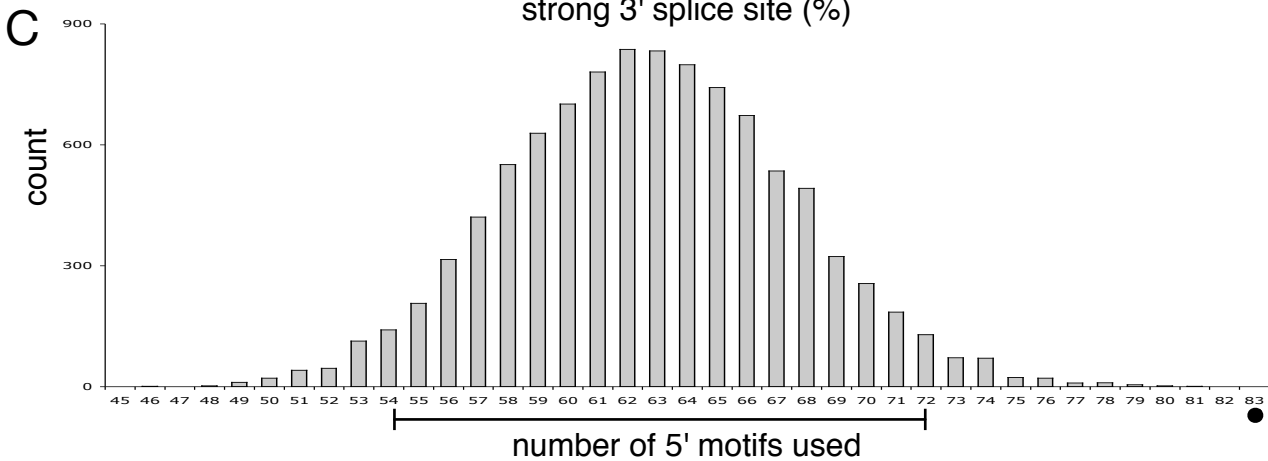

Supplement: Figure S11 — Resampling analysis of splice site usage. We resampled (with replacement) 307 from the set of 307 novel introns (black) and 307 from the set of all 50,836 introns (gray) to obtain a distribution of the proportion of introns that carry the most common motif at the 5′ (A) and at the 3′ (B). The observed values for novel (black dot) and all (gray dot) are shown below each graph with 95% CI taken from the distributions above. The observed values for novel are outside the 95% CI for the distribution for all introns. Resampling from the set of novel is not actually required to establish significance, but does give an indication of the variation within novel introns. (C) The 307 novel introns use 83 different motifs at the 5′ splice site (black dot), outside the distribution of values obtained by resampling (307 samples, 10,000 times) from all (393 different motifs, 50,836 introns), indicating that novel introns use a more diverse set of splice sites than expected (i.e. more rare/weak motifs). (0.04 MB PDF) [file pgen.1000819.s011.pdf]

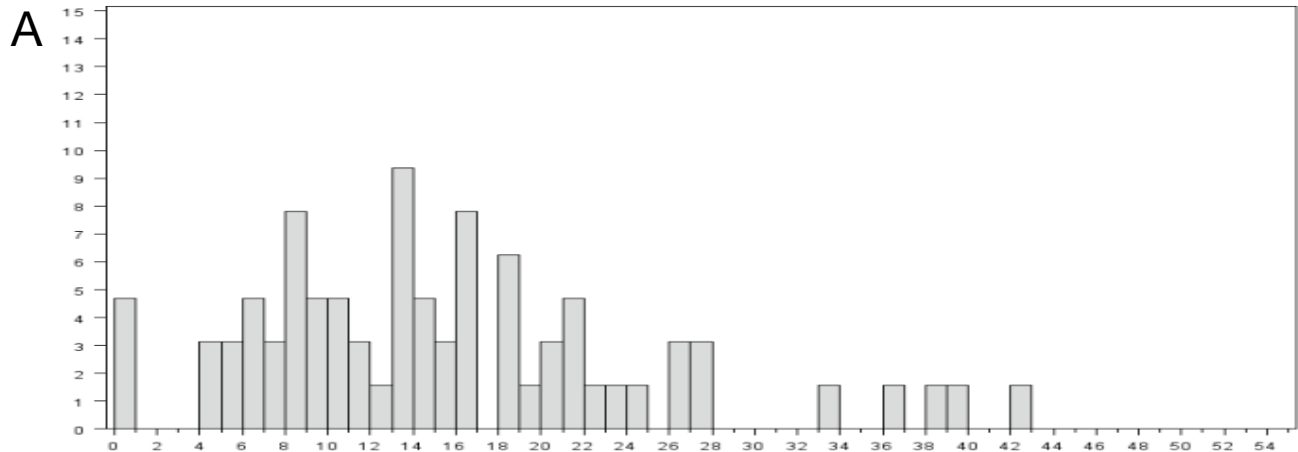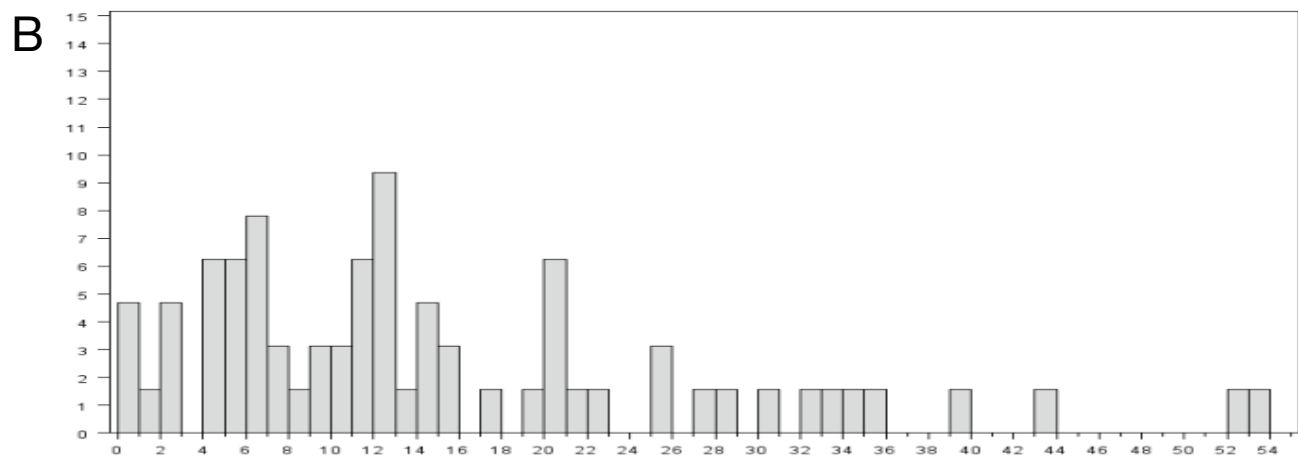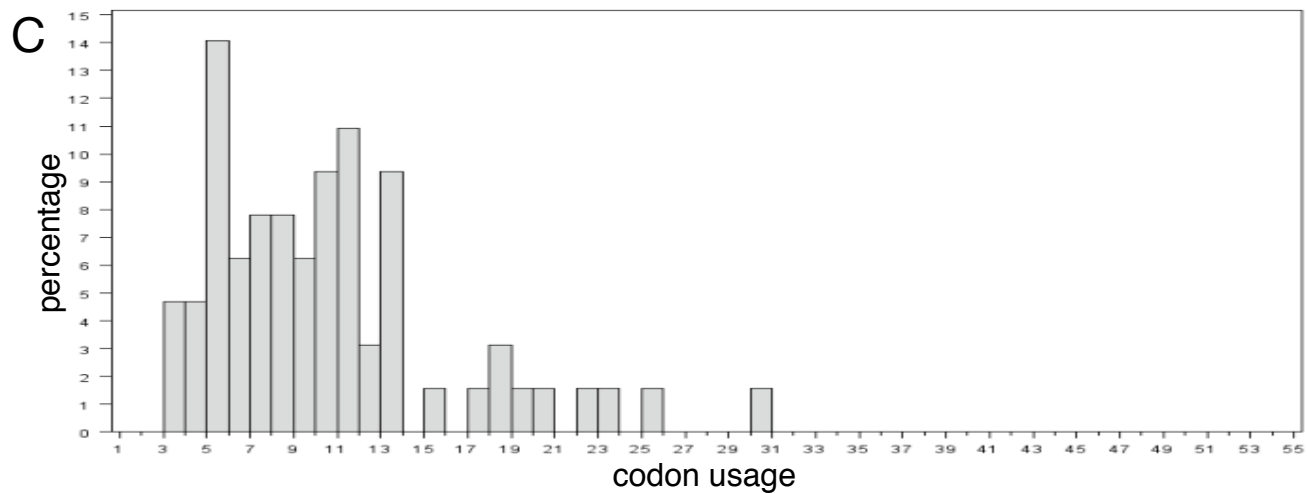

Supplement: Figure S12 — Novel introns show not codon usage bias. Distribution of codon usage values for all 64 codons for (A) all D. melanogaster genes (http://www.kazusa.or.jp/codon/cgi-bin/showcodon.cgi?species=7227) (B) the 180 insertions excluded form our set of novel introns and (C) the 307 novel introns. Spearman Correlation Coefficients indicate significant codon usage bias in insertions (0.57989, P<0.0001) further justifying their exclusion from our data set, but no bias within novel introns (0.01983, P = 0.8764). (0.08 MB PDF) [file pgen.1000819.s012.pdf]

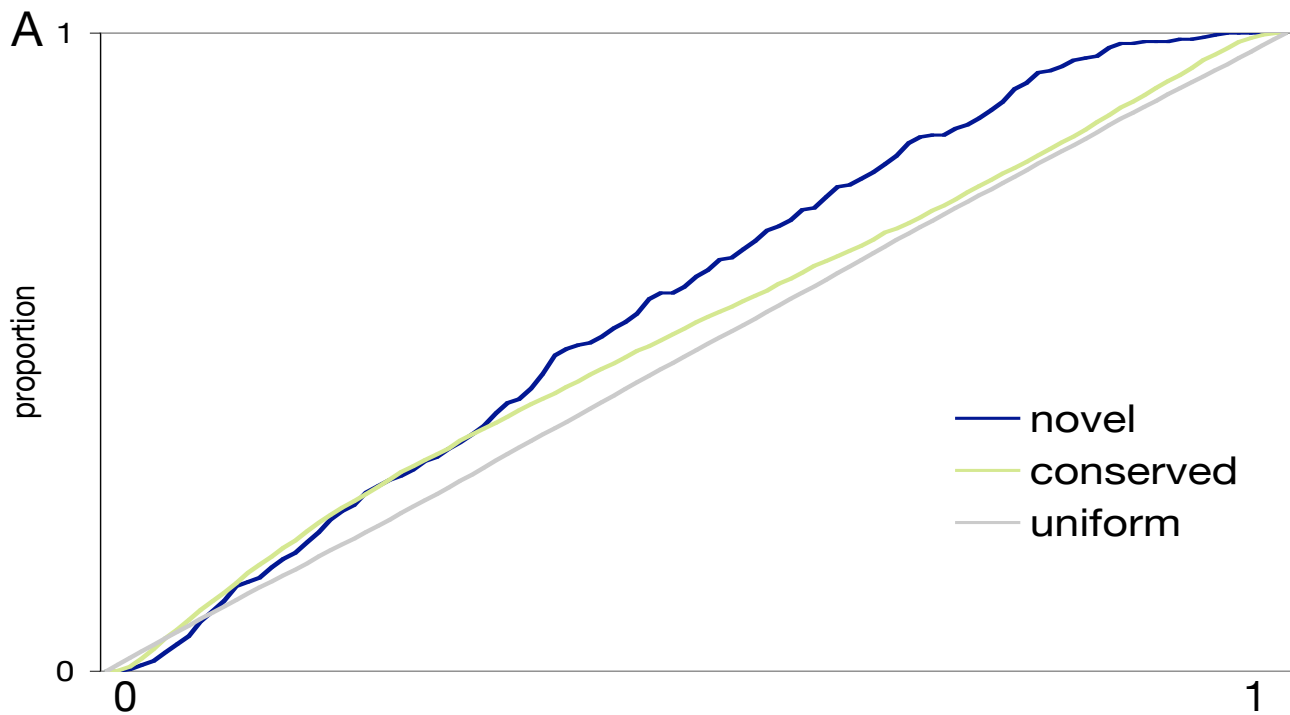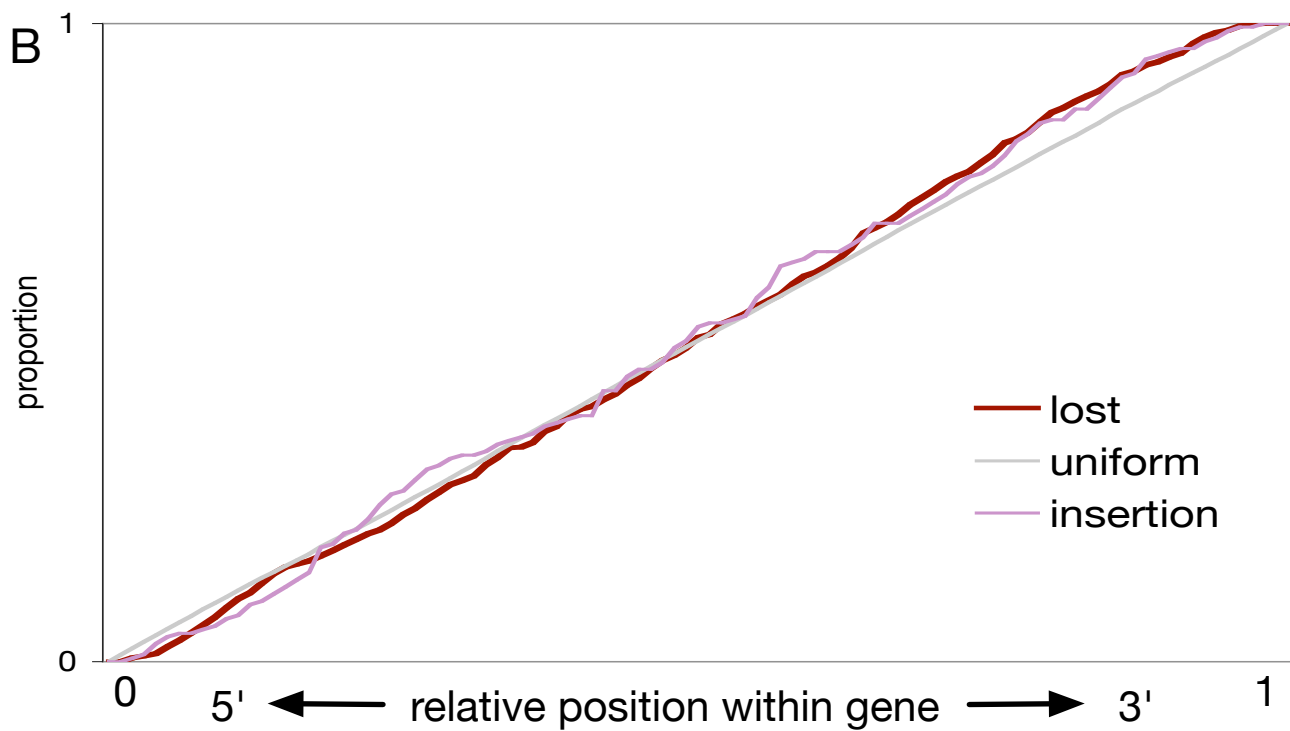

Supplement: Figure S13 — Novel introns are strongly biased towards the 5′ end of the gene. Empirical cumulative distribution of intron position across the gene for (A) novel and conserved introns, and (B) lost introns and insertions. Compared to a uniform distribution novel (X2 = 26.063, P<0.001) and conserved (X2 = 110.554, P<0.0001) both show a 5′ bias. This bias is stronger for novel introns (X2 = 7.273, P = 0.007). Lost introns (X2 = 1.309, P = 0.253) and insertions (X2 = 0.495, P = 0.482) do not differ from the uniform distribution. (0.04 MB PDF) [file pgen.1000819.s013.pdf]
