# Supplementary material for: Nonsense-Mediated Decay Enables Intron Gain in Drosophila
Source: PLoS Genet. 2010 Jan 22;6(1):e1000819. doi: 10.1371/journal.pgen.1000819 (PMC2809761; doi:10.1371/journal.pgen.1000819)
Supplement: Text S1 — Supplemental methods. (0.06 MB DOC) [file pgen.1000819.s014.doc]

**Text S1** - **Supplementary Methods**

**GeneWise limitations**

**(1)** **Extreme ends.** GeneWise becomes unreliable at the extreme ends of a gene when the amino acid conservation is weak [46], sometimes resulting in the false prediction of a long intron to improve the amino acid alignment (false positive intron gain). We manually scrutinised predicted novel introns at the extreme ends, removing those that did not have convincing sequence conservation in the extreme exon. GeneWise also becomes unreliable when a genuine intron is present at an extreme end of a gene (i.e. a short first exon), resulting in an abridged gene annotation. To remove this potential source of false intron loss, we excluded form our data set genes in which the first or last intron was missing from an orthologous partner. For the purpose of this study, if an ortholog contains the first and last intron from *D. melanogaster* it is considered a full length gene prediction.

**(2) intron splitting** occurs when GeneWise invents a very short exon within a very long intron (due to an AG-3n-GT motif). These events were easily identified within the GeneWise output as an exon absence in *D. melanogaster* (which in all cases was only 2-3 codons long) and were excluded.

**(3) intron sliding** (due either to amino acid *indels* shifting the intron position within the GeneWise alignment, or to the actual movement of an intron over short distances) appeared as adjacent intron gain and loss events. These cases where manually inspected and only those with convincing sequence conservation between the gain/loss position where retained in the final data set.

**(4)** Using the 2193 algorithm approximately 300 GeneWise jobs failed for each species (due to memory limitations). These genes were successfully re-submitted to the GeneWise 623 algorithm.

**False positives and false negatives**

Dealing with false negatives was straightforward; every intron that was absent only in a single species was manually scrutinised (loss events occurring at a node were considered real because of the independent prediction in more than one species). We considered 86 predicted intron loss events to be false, because the underlying intronic sequence was still present (but was of length 3n), potential splice sites were present (in several cases the 5' site was the non-canonical GC) and flybase (<http://flybase.org/>) predicted the intron (Sheet 8 in Dataset S1).

Dealing with potential false positive intron gains was less straightforward. Intron gain events either occurred at an internal node of the species tree or in a single species (a terminal leaf). For a gain to be identified at a node it must have be independently predicted in one or more neighbouring species, making the possibility of a false prediction less likely. Therefore, we considered all gains that occurred at a node to be genuine. Except where mentioned otherwise, we considered the *D. melanogaster* representative of each gain at a node (or *D. virilis* for the 4 gains shared with *D. mojavensis*). An excess of novel 3n PTC-free "introns" in the terminal leaves of the tree indicated that our approach was over zealous in the classification of large gaps in the GeneWise alignment as novel introns, a common limitation of gene structure prediction [47]. As the conclusions of this paper are based on a deficiency of 3n PTC-free novel introns we have been careful that all *bona fide* introns have been correctly included in our final data set and all false positives excluded. Furthermore, we show that our conclusion (deficiency of 3n PTC-free novel introns) hold even when we restrict our logistic regression analysis to the set of novel introns gained at a node from which no 3n PTC-free introns were excluded (discussed below).

We arrive at our final set of novel introns after experimental validation via (1) the EST database and (2) RT-PCR, and subsequent validation via (3) analysis of length, (4) intron phase, (5) codon usage bias, (6) 5' bias and (7) "splice sites" strength.

**(1) EST sequences -** We probed the EST sequence databases of each species using BLAST to confirm the splicing of each putative novel intron. Using 100 bp of exonic sequence flanking the intron (concatenated from each side to mimic the spliced transcript) a BLASTn (default parameters) hit (100% identity) indicated a genuine intron (Hits 60-100 bp were inspected manually to confirm a real splicing event in an incomplete EST or EST with variant sequence). Additionally, the full intronic sequence was used to confirm a lack of splicing. A BLAST hit with both approaches indicated alternative splicing (intron retention). For intron gain events occurring at nodes within the species tree (and therefore present in more than one species) each copy of the novel intron was tested separately (subsequent numbers consider each gain only once).

We identified informative ESTs for 175 putative novel introns (Sheet 7 in Dataset S1). The remainder are either not expressed sufficiently to occur in an EST database or are in a species with poor or no EST coverage. For introns that disrupted the open reading frame (ORF) 100% of ESTs confirmed splicing (150/150 - including 7 cases of intron retention). Based on this, we included all ORF-disrupting novel introns in our final data set.

ESTs confirmed the splicing of 8 3n PTC-free introns and the non-splicing of 17 3n PTC-free insertions. We included these 8 introns in our final data set and excluded both the 17 events and the remaining 163 without EST support (we refer to them as insertions). Of course this raised the likely possibility that some of the remaining 163 insertions are in fact genuine introns.

To explore this possibility we used **(2) RT-PCR**. We generated oligo-dT cDNA from pooled RNA extracted from multiple life stages and both sexes (after DNase treatment) from *D. ananassae, D. pseudoobscura* and *D. willistoni.* We confirmed the splicing of 11 out of 11 ORF-disrupting novel introns for which we did not previously find ESTs (including 1 new case of intron retention). We confirmed the splicing of 4 out of 4 3n PTC-free introns for which we had already found ESTs. We obtained RT-PCR products for a further 39 3n PTC-free events (for which no EST was previously identified) and zero cases showed splicing. Based on this result we speculated that very few (possibly zero) of the remaining 124 3n PTC-free insertions are genuine introns. Instead, these events represent insertions within exonic sequence that are long enough to confound the GeneWise algorithm and therefore, were exclude from the data set. We support this assumption by demonstrating that these 180 excluded insertions differ from novel (and conserved) introns in all 5 characteristics discussed below.

**(3) Insertions are longer than novel introns** Novel introns are significantly shorter than phylogenetically conserved introns (median 63 bp vs. 66 bp. Kruskal-Wallis test χ^2^=25.4, P<0.001). Insertions are longer than novel introns (median 84 bp. Kruskal-Wallis Test χ^2^=51.4206, P<0.0001). Novel introns also show a larger size range suggesting that they are free to evolve in length (both down and up), whereas insertions are constrained by their initial length.

**(4) Insertions show an unusual intron phase distribution**, phase 0 = 23.3%, phase 1 = 56.1% and phase 2 = 20.6% which differs significantly from the 60:20:20 of novel introns (χ^2^=166.8225, Pr <0.0001).

**(5) Insertions show significant codon usage bias.** The correct reading frame for each insertion was considered using the phase information from the GeneWise output and the count of each codon was summed across all insertions. The Spearman rank correlation between codon usage of inserts and the reported codon usage bias for *D. melanogaster* was significant (0.57989, P<0.0001, N = 64) (Figure S12). Our approach to identifying codon usage bias is further discussed below. This result, in addition to the experimental results above, are the strongest support for these events as protein coding, justifying their exclusion from our data set.

**(6) Insertions do not have a 5' bias.** Both novel and conserved introns are biased towards the 5' end of the transcript. Insertions do not differ from a uniform distribution across the gene (Mann-Whitney test, see Figure S13).

**(7) Insertions have weaker "splice sites" and change the coding sequence length more frequently**. Of the 180 insertions only two (1.1%) have the strong canonical 5' splice site, compared to 19% for novel and 43% for all introns. Furthermore, if they really underwent splicing only 38% would leave the coding sequence unchanged, in contrast to the 87% of novel introns. It is also notable that all 180 insertions were identified in the terminal branches of the species tree (i.e. only in a single species). Therefore, we consider these 180 events to be large protein coding insertions and use them in support of our assertion that random insertions within coding regions present a viable origin for novel introns. This is because they are large, common, carry at least the minimal sequence requirements for *in silico* intron prediction and in no case was an homologous origin identified elsewhere within the genome via BLAST (data not shown). Therefore, we report after extensive *in silico* and experimental validation 307 novel introns amongst 284 genes across nine *Drosophila* species.

**Testing support for Reverse Splicing and Mobile Element based intron gain**

Both models require an homologous parental copy of the novel intron elsewhere within the genome that would initially show complete sequence identify. We conducted a comprehensive search for such evidence using BLAST against the *Drosophila* transposable element database and the whole genome of each appropriate species (including the unassembled U-chromosome component). The low complexity filter was not used and all significant hits (E-value <10^-5^) were manually inspected. In a single case, a gene had undergone duplication after gain of the novel intron, hence we observed almost complete conservation of both the novel intron and the flanking coding sequence. All the remaining significant hits fell in low complexity repeats and could not be detected with the low complexity filter option. The small size of most novel introns argues against mobile elements (which are usually several Kb in length) as their origin.

Biochemical demonstration that the catalytic removal of introns via the spliceosome is reversible [48] has caused a resurgence in the reverse splicing model [49], leading to the prediction that both reverse splicing intron gain and cDNA recombination intron loss proceed via a common mechanism. We tested the expectation that this would produce an excess of adjacent intron gain and loss events. We identified 8 novel introns that possibly occurred concurrently with an intron loss event. 4 cases were adjacent, 4 were separated by a third conserved intron (Sheet 4 in Dataset S1). Finally, the strong 5' bias we observe in novel introns is not consistent with a mechanism dependent on the action of reverse transcriptase (which is expected to give a 3' bias). Hence, despite the large number of novel and lost introns we report here, we do not find support for reverse splicing as a common mechanism of intron gain.

**Testing support for Tandem Duplication based intron gain (Direct repeats)**

As above (for testing reverse splicing) we used BLAST of each novel against the parental gene and asked for any hits not aligning perfectly with the intron. We found no informative hits. A further attempt to find the signature of tandem duplication was made with Dotplots (constructed in R with *seqinr*) [50] with a match and window size of 6/10, 8/10 and 8/8 and inspected manually for all 307 novel introns. On initial examination only a single example of "tandem duplication" was identified (Figure S3) because we were looking for extended tracts of sequence identity, not short direct repeats. On re-examination of the 8/8 dotplots we found 6 more genes with direct repeats (Figure S4-S9) and a further 8 in which one or both of the repeats did not cover a splice site (data not shown). Examination of 6/6 dotplots gave many more putative examples (data not shown).

**Testing support for Intronisation based intron gain**

Intronisation is expected to cause the loss of amino acid sequence equal to the length of the initial intron [51] (which may subsequently change length) and would appear as a gap in the GeneWise alignment. We looked for gaps of any length in the alignment flanking (±5 amino acids) novel introns. 13% of novel introns showed a gap and each case was manually inspected to establish the true length of the gap. In almost all cases this length was only 1-3 amino acids, meaning that 1-3 amino acids have been gained or lost form the coding sequence at the site of the intron. The most likely explanation is the use of cryptic slice sites in close proximity to the new sequence (in a manner analogous to tandem splice sites^28^). This result and the lack of codon usage bias (discussed below) supports the conclusion that intronisation is not a common mechanism for intron gain in *Drosophila*, despite being so in *C. elegans* [51].

**The prevalence of PTC-free novel introns**

To identify PTCs within introns we predicted the maximum expected amino acids length for each putative intron using intron length and phase (extracted from GeneWise). Each intronic sequence was then translated using virtual ribosome [52] in the appropriate reading frame (phase 0, frame 1; phase 1, frame 3; phase 2, frame 2) and a PTC was inferred if the product was shorter than expected. The probability of having an inframe PTC was contrasted for all combinations of novel vs. conserved introns in all 3n classes applying a logistic regression *proc logistic* (SAS, 2008)*,* correcting for the fixed categorical effect of phase, a combined effect of 3n class (3n vs. 3n+1 and 3n+2) and whether an intron in novel or conserved, and fitting intron length as covariate.

In order to confirm that our finding were robust and not an artifact of the inappropriate exclusion of 3n PTC-free introns during the validation process, we restricted our logistic regression to include only the 133 novel introns that have been gained at a node of the species tree. As every novel intron in this set has been independently predicted in two or more species all 3n PTC-free introns were included as genuine. Hence, this restricted data set is not influenced by the possible incorrect exclusion of 3n PTC-free introns/insertions that may influence the analysis of all 307 novel introns. Despite a loss of power due to the reduced number of novel introns, the contrast between novel 3n and novel 3n+1&2 remained significant (Odds ratio 3.854, 95% CI 1.053-14.110).

In all our analysis thus far we considered only the *D. melanogaster* representative of each novel intron gained at a node. For example, a novel intron at node 4 is present in 5 species and therefore has 5 independent lengths and PTC statuses (in all cases phase was conserved). So while we identified 307 intron gain events, we can analyse 667 intronic sequences. Re-running our logistic regression on this expanded data containing all 667 sequences also returned a significant difference between novel 3n and novel 3n+1,3n+2 (Odds ratio 1.916, 95% CI 1.186-3.097). In both cases above, novel introns return a higher odds ratio than conserved introns (odds ratio 1.646). Therefore, the deficiency of 3n PTC-free introns in the genome and our observation that this deficit is stronger for novel introns is robust.

Statistical analyses were performed with the software package SAS 9.2 (SAS Institute Inc., 2008) or Excel (Microsoft). The level of significance was set to P<0.05. Correction for multiple testing (where appropriate) was carried out using Tukey-Cramer correction [53]. Splice site motifs were manipulated, counted and resampled using in house *Python* script (available at http://i122server.vu-wien.ac.at/Drosophila_annotation/).

**Codon usage bias, intron retention rates and 5' bias of novel introns**

Existing codon usage bias calculators delimit the coding sequence based on start and stop codons rendering them unhelpful for analysing intronic sequence (which do not carry start codons and may contain multiple stop codons), therefore, we determined codon usage by first counting each codon (accounting for the phase of each intron) and dividing by the total number of codons. This allowed direct comparison between our 180 insertions and the reported codon usage counts for *D. melanogaster* (<http://www.kazusa.or.jp/codon/>), applying a Spearman rank correlation across all 64 pairs of codons (0.57989, P<0.0001) (Figure S12).

In contrast, the Spearman correlation coefficient between novel introns and codon usage reported for *D. melanogaster* was 0.01983 (P=0.8764). As the high AT base composition of introns may distort the codon usage pattern within novel introns we compared them to the codon usage calculated after randomly assigning a phase to each novel intron (33% into each phase). This gave a Spearman correlation coefficient of 0.97072 (P<0.0001) indicating that base composition does account for codon frequency within novel introns.

Our RT-PCR and EST library search identified 8/307 novel introns that undergo intron retention. In all cases the retained intron was the minor isoform. We repeated this BLAST approach (discussed above) to identify intron retention events within a set of 9,895 introns in *D. melanogaster* and identified 531 intron retention events.

To assess relative 5'–3' distribution of each class of introns (novel, conserved and lost) within a gene we compared introns within each class to a simulated uniform distribution, and the class of novel introns to the class of conserved introns via Mann-Whitney tests (P*roc Npar1way* option *wilcoxon)*. Furthermore, we produced empirical distribution function graphs (P*roc Npar1way* option *plots=edfplot*) for each class and the simulated uniform distribution for graphical presentation of the data (Figure S13).

46. Birney E, Clamp M, Durbin R (2004) GeneWise and Genomewise. Genome Res 14: 988-995.

47. Roy SW, Penny D (2007) Intron length distributions and gene prediction. Nucleic Acids Res 35: 4737-4742.

48. Tseng CK, Cheng SC (2008) Both catalytic steps of nuclear pre-mRNA splicing are reversible. Science 320: 1782-1784.

49. Roy SW, Irimia M (2009) Mystery of intron gain: new data and new models. Trends Genet 25: 67-73.

50. R Development Core Team , A Language and Environment for Statistical Computing. http://www.R-project.org (2009).

51. Irimia M, Rukov JL, Penny D, Vinther J, Garcia-Fernandez J, et al. (2008) Origin of introns by 'intronization' of exonic sequences. Trends Genet 24: 378-381.

52. Wernersson R (2006) Virtual Ribosome--a comprehensive DNA translation tool with support for integration of sequence feature annotation. Nucleic Acids Res 34: W385-388.

53. Kramer CY (1965) Extension of Multiple Range Tests to Group Means with Unequal Numbers of Replications. Biometrics 12.
